# Role of genetic polymorphisms of ion channels in the pathophysiology of coronary microvascular dysfunction and ischemic heart disease

**DOI:** 10.1007/s00395-013-0387-4

**Published:** 2013-09-26

**Authors:** Francesco Fedele, Massimo Mancone, William M. Chilian, Paolo Severino, Emanuele Canali, Suzanna Logan, Maria Laura De Marchis, Maurizio Volterrani, Raffaele Palmirotta, Fiorella Guadagni

**Affiliations:** 1 Department of Cardiovascular, Respiratory, Nephrology, Anesthesiology and Geriatric Sciences, Sapienza University of Rome, Umberto I Policlinic, Viale del Policlinico 155, 00161 Rome, Italy; 2 Department of Integrative Medical Sciences, Northeastern Ohio Universities College of Medicine, Rootstown, OH, USA; 3 Department of Advanced Biotechnologies and Bioimaging, IRCCS San Raffaele Pisana, Rome, Italy; 4 Source Cardiovascular Research Unit, Department of Medical Sciences, Centre for Clinical and Basic Research, IRCCS San Raffaele Pisana, Rome, Italy

**Keywords:** Ion channels, Genetic polymorphisms, Coronary microcirculation, Endothelium, Atherosclerosis, Ischemic heart disease

## Abstract

Conventionally, ischemic heart disease (IHD) is equated with large vessel coronary disease. However, recent evidence has suggested a role of compromised microvascular regulation in the etiology of IHD. Because regulation of coronary blood flow likely involves activity of specific ion channels, and key factors involved in endothelium-dependent dilation, we proposed that genetic anomalies of ion channels or specific endothelial regulators may underlie coronary microvascular disease. We aimed to evaluate the clinical impact of single-nucleotide polymorphisms in genes encoding for ion channels expressed in the coronary vasculature and the possible correlation with IHD resulting from microvascular dysfunction. 242 consecutive patients who were candidates for coronary angiography were enrolled. A prospective, observational, single-center study was conducted, analyzing genetic polymorphisms relative to (1) NOS3 encoding for endothelial nitric oxide synthase (eNOS); (2) *ATP2A2* encoding for the Ca^2+^/H^+^-ATPase pump (SERCA); (3) *SCN5A* encoding for the voltage-dependent Na^+^ channel (Nav1.5); (4) *KCNJ8* and *KCNJ11* encoding for the Kir6.1 and Kir6.2 subunits of K-ATP channels, respectively; and (5) *KCN5A* encoding for the voltage-gated K^+^ channel (Kv1.5). No significant associations between clinical IHD manifestations and polymorphisms for SERCA, Kir6.1, and Kv1.5 were observed (*p* > 0.05), whereas specific polymorphisms detected in eNOS, as well as in Kir6.2 and Nav1.5 were found to be correlated with IHD and microvascular dysfunction. Interestingly, genetic polymorphisms for ion channels seem to have an important clinical impact influencing the susceptibility for microvascular dysfunction and IHD, independent of the presence of classic cardiovascular risk factors.

## Introduction

Historically, in the interrogation of altered vascular function in patient with ischemic heart disease (IHD), scientists have focused their attention on the correlation between endothelial dysfunction and atherosclerosis [11, 53, 65, 67]. However, the endothelium-independent dysfunction in coronary microcirculation and its possible correlations with atherosclerotic disease and myocardial ischemia have not been extensively investigated. In normal conditions, coronary blood flow regulation (CBFR) is mediated by several different systems, including endothelial, nervous, neurohumoral, myogenic, and metabolic mechanisms [2, 10, 14, 15, 63, 64, 69]. Moreover, physiologic CBFR depends also on several ion channels, such as ATP-sensitive potassium (KATP) channels, voltage-gated potassium (Kv) channels, voltage-gated sodium (Nav) channels, and others. Ion channels regulate the concentration of calcium in both coronary smooth muscle and endothelial cells, which in turn modulates the degree of contractile tone in vascular muscle and the amount of nitric oxide that is produced by the endothelium, respectively. In this context, ion channels play a primary role in the rapid response of both the endothelium and vascular smooth muscle cells of coronary arterioles to the perpetually fluctuating demands of the myocardium for blood flow [5, 6, 13, 18, 33, 45, 46, 51, 52, 61, 73, 75].

Despite this knowledge, there still exists an important gap about the clinical relevance and causes of microvascular dysfunction in IHD. By altering the overall regulation of blood flow in the coronary system, microvascular dysfunction could alter the normal distribution of shear forces in large coronary arteries, thus promoting atherosclerosis. On the other hand, proximal coronary artery stenosis could contribute to microvascular dysfunction [29, 60]. Because ion channels play such a critical role in microvascular endothelial and smooth muscle function, we hypothesized that alterations of coronary ion channels could be the *primum movens* in a chain of events leading to microvascular dysfunction and myocardial ischemia, independent of the presence of atherosclerosis. Therefore, the objective of our study was to evaluate the possible correlation between IHD and single-nucleotide polymorphisms (SNPs) for genes encoding several regulators involved in CBFR, including ion channels acting in vascular smooth muscle and/or endothelial cells of coronary arteries.

## Methods

In this prospective, observational, single-center study, 242 consecutive patients admitted to our department with the indication to undergo coronary angiography were enrolled. All patients matched inclusion (age >18; suspected or documented diagnosis of acute coronary syndrome or stable angina with indication(s) for coronary angiography, in accordance with current guidelines [36, 68], and the same ethno-geographic Caucasian origin) and exclusion criteria (previous allergic reaction to iodine contrast, renal failure, simultaneous genetic disease, cardiogenic shock, non-ischemic cardiomyopathy). All patients signed an informed consent document prior to participation in the study, which included acknowledgement of the testing procedures to be performed (i.e., coronary angiography; intracoronary tests; genetic analysis, and processing of personal data). The study was approved by the Institution’s Ethics Committee. All clinical and instrumental characteristics were collected in a dedicated database.

### Study design

- Standard therapies were administered, according to current guidelines [36, 68].
- In all patients, an echocardiography evaluation before and after coronary angiography was performed.
- According to standard clinical practice, coronary angiography was performed using radial artery or femoral artery Judkins approach via sheath insertion.
- In patients showing normal epicardial arteries, intracoronary functional tests were performed through Doppler flow wire to evaluate both endothelium-dependent microvascular function [via intracoronary (IC) infusion of acetylcholine (2.5–10 μg)] and non-endothelium-dependent microvascular function [via IC infusion of adenosine (5 μg)] [31].
- In all enrolled patients, a peripheral blood sample for genetic analysis was taken.

**Fig. 1:**
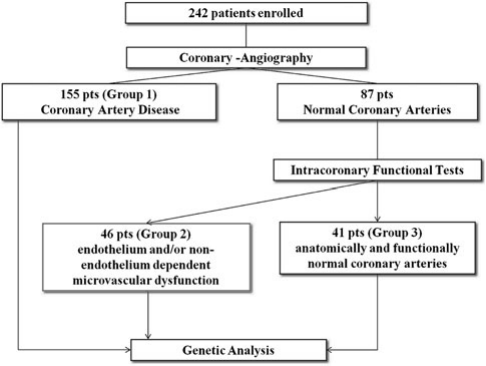
Study design: 242 consecutive not randomized patients matching inclusion and exclusion criteria were enrolled. In all patients, coronary angiography was performed, according to current ESC/ACC/AHA guidelines. In patients with angiographically normal coronary artery, intracoronary functional tests were performed. In 242 patients (155 with coronary artery disease, 46 patients with microvascular dysfunction, endothelium and/or non-endothelium dependent, and 41 patients with anatomically and functionally normal coronary arteries) genetic analysis was performed

On the basis of the coronary angiography and the intracoronary functional tests, the 242 patients were divided into three groups (see also Fig. 1).

- Group 1: 155 patients with anatomic coronary alteration (comprising patients with acute coronary syndrome and chronic stable angina).
- Group 2: 46 patients with functional coronary alteration [normal coronary arteries as assessed by angiography, and microvascular dysfunction defined as coronary flow reserve (CFR) < 2.5 after IC infusion of acetylcholine and adenosine].
- Group 3: 41 patients with anatomically and functionally normal coronary arteries as assessed by angiography and with normal functional tests (CFR ≥ 2.5 after intracoronary infusion of acetylcholine and adenosine) (Fig. 1).

### Genetic analysis

In conformity with the study protocol, ethylenediaminetetraacetic acid (EDTA) whole blood samples were collected according to the international guidelines reported in the literature [48]. Samples were transferred to the Interinstitutional Multidisciplinary BioBank (BioBIM) of IRCCS San Raffaele Pisana (Rome) and stored at −80 °C until DNA extraction.

Bibliographic research by PubMed and web tools OMIM (http://www.ncbi.nlm.nih.gov/omim), Entrez SNP (http://www.ncbi.nlm.nih.gov/snp), and Ensembl (http://www.ensembl.org/index.html) were used to select variants of genes involved in signaling pathways related to ion channels and/or previously reported to be associated with microvascular dysfunction and/or myocardial ischemia and/or diseases correlated to IHD, such as diabetes mellitus.

Polymorphisms for the following genes were analyzed: *NOS3* (endothelial nitric oxide synthase, eNOS), *ATP2A2* (Ca^2+^/H^+^-ATPase pump, SERCA2), *SCN5A* (voltage-dependent Na^+^ channel, Nav1.5), *KCNJ11* (ATP-sensitive K^+^ channel, Kir6.2 subunit), *KCNJ8* (ATP-sensitive K^+^ channel, Kir6.1 subunit) and *KCNA5* (voltage-gated K^+^ channel, Kv1.5). In particular, we completely analyzed by direct sequencing exon 3 of *KCNJ8* (Kir6.1 subunit), which includes eight SNPs, as well as the whole coding region of *KCNA5* (Kv1.5 channel), which includes 32 SNPs and four previously described variants [26, 47, 71, 72]. We also examined the whole coding region of *KCNJ11* (Kir6.2 subunit), for which sequence variants have been described [26, 28]. All SNPs and sequence variants analyzed—a total of 62 variants of 6 genes—are listed in Table 1.

**Table 1:** All SNPs and sequence variants analyzed in the study are listed

Protein	Gene	OMIM	SNP ID	N change	Amino acid change	Primers	Ta °C
eNOS	*NOS3*	163729	rs1799983	G-T	Glu298Asp	5′-CATGAGGCTCAGCCCCAGAAC-3′	60
						5′-AGTCAATCCCTTTGGTGCTCAC-3′	
SERCA	*ATP2A2*	108740	rs56243033	G-A	Ala724Ala	5′-TGAACGATGCTCCTGCTCTG-3′ 5′-TGGGACGAGATGAGGTAGCG-3′	60
			rs12312588	G-A	Leu734Leu		
Nav1.5	*SCN5A*	600163	rs6599230	A-G	Ala29Ala	5′-CCTCTGCTCCATTGACAAGG-3′ 5′-GCAGCCATCGAGAAGCG-3′	60
			rs1805124	A-G	His558Arg	5′-TATGAAGCCACGTTCCAGCC-3′ 5′-CCATTGCAGTCCACAGTGC-3′	
Kir6.1	*KCNJ8* ^*a*^	600935	rs11046182	A-G	Intron 2	5′-TCGAGATGAAACTGTTCCACC-3′ 5′-TGCCATCTTATATGGGCATGGC-3′	60
			rs74069151	A-G	Intron 2	5′-AAGAGGTAGGCTGGATAATATCG-3′ 5′-ATTTCCAAATAATGTTTGACCATTC-3′	
			rs77763829	A-G	Ala171Ala	5′-AAGTTGGCTAGTCTTTCTGCAAGC-3′ 5′-TTCTTGACCACCTGGATGCGCAC-3′	
			rs16924297	T-C	Leu265Leu	5′-AAGCTGTGCTTCATGTTCCG-3′ 5′-TTAACAGTGTTGCCAAATTTGG-3′	60
			rs34093632	G-A	Leu285Leu		
			rs35941868	C-T	Ser312Ser		
			rs34130387	delC	delC315		
			rs34811413	T-C	Val334Ala		
			rs34951653	delC	delC391	5′-TGTCCATTGTGACTGAGGAAGAAGG-3′ 5′-AACAGACTCATTTCTTGACC-3′	60
			rs72554071	C-T	Ser422Leu		
			rs7310043	T-A	3′UTR	5′-TTGTTTCATACATGTAGAATTCGC-3′ 5′-TTATGGTATGTCAGGCTTAGATTG-3′	60
			rs7309783	T-A	3′UTR		
			rs7295420	A-G	3′UTR		
Kir6.2	*KCNJ11* ^*a*^	600937	rs5219	A-G	Lys23Glu	5′-GTGGAGGTAAGGAAGAGTCTGG-3′ 5′-AGACGAGAAGGAGTGGATGC-3′	59
			rs140636367 rs5218	G-A C-T	Ser118Ser Ala190Ala	5′-AAGTGGCCACACACATTGC-3′ 5′-TCAATGACATGGTAGATGATCAGC-3′	59
			rs5216	C-G	Leu267Leu	5′-TGGACATCCCCATGGAGAAC-3′ 5′-CTTGTAACACCCTGGATGAGC-3′	59
			rs1800467	C-G	Leu270Val		
			rs5215	G-A	Val337Ile		
			rs8175351	G-A	Lys381Lys		
			rs41282930	C-G	Ser385Cys		
Kv1.5	*KCN5A* ^*b*^	176267	rs61737395	G-T	Gly31Val	5′-TTCTTGACGTCAGGGCCAAGCG-3′ 5′-CCGGAGATGTTGATGTGGACG-3′	60
			rs71584818	A-T	Glu33Val		
			rs71581015	27 bp del	Ser62_Asp72 del		
			rs71541953	C-T	Pro73Ser		
			rs71537801	G-A	Arg87Gln		
			ND	C-T	Pro91Leu		
			rs17215395	C-T	Ala115Val		
			rs45504599	C-T	Ser127Ser	5′-ACCAGGCTCTGGGCACGGCG-3′ 5′-TGGAGATGAGGATAACCAAGACCG-3′	60
			rs1056462	G-C	Gly128Gly		
			rs1056463	T-A	Leu138Gln		
			rs41276730	C-A	Leu185Met		
			rs12720444	C-T	Asn190Asn		
			rs12720443	G-C	Leu205Leu		
			rs35853292	G-C	Glu211Asp		
			rs77281462	C-T	Arg212Cys		
			rs3197074	C-G	Arg214Gly		
			rs1056464	C-T	Pro228Ser		
			rs55699243	T-G	Leu238Leu		
			rs55874756	T-C	Ser248Pro		
			rs55958438	C-A	Arg250Arg		
			rs45618444	C-G	Ala251Gly		
			rs17215409	C-T	Pro307Ser	5′-TTATCTTCGAGTATCCGGAGAGC-3′ 5′-TGGAGGCCTGCAAGGTCTTG-3′	60
			rs17215402	C-T	Pro310Leu		
			rs72546671	C-T	Leu340Leu		
			rs61753194	T-A	Val341Glu		
			rs35130466	Ins C	InsC367		
			ND	G-T	Glu375Ter		
			rs2359641	T-C	Gly383Gly		
			rs76708779	G-A	Gly384Arg		
			rs17221805	G-A	Leu499Leu	5′-GTGTTCCGCATCTTCAAGC-3′ 5′-TAGATATCCATGTTCAGCAAGCC-3′	60
			ND	C-T	Thr527Met		
			rs71582899	G-A	Pro532Pro		
			rs71581016	G-A	Arg554Gln		
			rs71581017	G-T	Gly568Val		
			ND	C-T	Ala576Val		
			rs12720445	G-A	Arg578Lys		

*ND* not determined, *eNOS* endothelial nitric oxide synthase, encoded by *NOS3* gene; *SERCA* sarco/endoplasmic reticulum Ca^2+^-ATPase, encoded by *ATP2A2* gene; *Nav1.5* voltage-gated Na^+^ channel, encoded by *SCN5A* gene; *Kir6.1 and Kir6.2* inward rectifying subunits of ATP-sensitive K^+^ channel, encoded by *KCNJ8* and *KCNJ11* genes, respectively; *Kv1.5* voltage-gated K^+^ channel, encoded by *KCN5A* gene. *N change* nucleotide change; *Ta* annealing temperature (°C), *SNP ID* Reference single-nucleotide polymorphism ID, see also http://www.ncbi.nlm.nih.gov/snp/; *OMIM#* Online Mendelian Inheritance in Man, see also http://www.ncbi.nlm.nih.gov/omim/

^a^Complete coding exon 3

^b^Whole coding region

DNA was isolated from EDTA anticoagulated whole blood using the MagNA Pure LC instrument and the MagNA Pure LC total DNA isolation kit I (Roche Diagnostics, Mannheim, Germany) according to the manufacturer’s instructions. Standard PCR was performed in a GeneAmp PCR System 9700 (Applied Biosystems, CA) using HotStarTaq Master Mix (HotStarTaq Master Mix Kit, QIAGEN Inc, CA). PCR conditions and primer sequences are listed in Table 1. In order to exclude pre-analytical and analytical errors, all direct sequencing analyses were carried out on both strands using Big Dye Terminator v3.1 Cycle Sequencing kit (Applied Biosystems), run on an ABI 3130 Genetic Analyzer (Applied Biosystems), and repeated on PCR products obtained from new nucleic acid extractions. All data analyses were performed in a blind fashion.

### Statistical analysis

This report, intended as pilot study, is the first to compare the prevalence of SNPs in genes encoding several effectors (including ion channels) involved in CBFR between these groups of patients. For this reason, no definite sample size could be formally calculated to establish a power analysis. However, assuming a 15 % prevalence of normal macrovascular and microvascular coronary findings in unselected patients undergoing coronary angiography, we estimated that a sample size of at least 150 patients could enable the computation of two-sided 95 % confidence intervals for such prevalence estimates ranging between −5.0 and +5.0 %.

The significance of the differences of observed alleles and genotypes between groups, as well as analysis of multiple inheritance models (co-dominant, dominant, recessive, over-dominant and log-additive) for SNPs were also tested using a free web-based application (http://213.151.99.166/index.php?module=Snpstats) designed from a genetic epidemiology point of view to analyze association studies. Akaike Information Criterion (AIC) was used to determine the best-fitting inheritance model for analyzed SNPs, with the model with the lowest AIC reflecting the best balance of goodness-of-fit and parsimony. Moreover, the allelic frequencies were estimated by gene counting, and the genotypes were scored. For each gene, the observed numbers of each genotype were compared with those expected for a population in Hardy–Weinberg (HW) equilibrium using a free web-based application (http://213.151.99.166/index.php?module=Snpstats) [59]. Linkage disequilibrium coefficient (D′) and haplotype analyses were assessed using the Haploview 4.1 program. Statistical analysis was performed using SPSS software package for Windows v.16.0 (SPSS Inc., Chicago, IL).

All categorical variables are expressed as percentages, and all continuous variables as mean ± standard deviation. Differences between categorical variables were analyzed by Pearson’s *χ* ^2^ test. Given the presence of three groups, differences between continuous variables, including the number of SNPs tested, were calculated using one-way ANOVA; a post-hoc analysis with Bonferroni correction was made for multiple comparisons.

Univariate and multivariate logistic regression analyses using enter method were performed to assess the independent impact of genetic polymorphisms on coronary artery disease and microvascular dysfunction, while adjusting for other confounding variables. The following parameters were entered into the model: age, male gender, type 2 diabetes mellitus (T2DM), systemic arterial hypertension, dyslipidemia, smoking status, and family history of myocardial infarction (MI). Only variables with a *p* value <0.10 after univariate analysis were entered into the multivariable model as covariates. A two-tailed *p* < 0.05 was considered statistically significant.

### Definition of cardiovascular risk factors

Patients were classified as having T2DM if they had fasting levels of glucose of >126 mg/dL in two separate measurements or if they were taking hypoglycemic drugs. Systemic arterial hypertension was defined as systolic blood pressure >140 mmHg and diastolic blood pressure >90 mmHg in two separate measurements or if the patient was currently taking antihypertensive drugs. Dyslipidemia was considered to be present if serum cholesterol levels were >220 mg/dL or if the patient was being treated with cholesterol-lowering drugs. Family history of MI was defined as a first-degree relative with MI before the age of 60 years.

## Results

Sixty-two polymorphisms distributed among six genes coding for nitric oxide synthase, the SERCA pump, and ion channels were screened for sequence variations using PCR amplification and direct DNA sequencing analysis in the population of 155 patients with CAD (group 1), 46 patients with microvascular dysfunction (group 2), and 41 patients with normal coronary arteries and normal endothelium-dependent and endothelium-independent vasodilation (group 3). In Group 3, the genotype distribution of SNP rs5215 (Kir6.2/*KCNJ11*) moderately deviates from the HW equilibrium (*p* = 0.05). In Group 1 (CAD), the polymorphism rs6599230 of Nav1.5/*SCN5A* showed deviation from HW equilibrium (*p* = 0.017). The genotypic distribution of rs1799983 polymorphism for eNOS/*NOS3* is inconsistent with the HW equilibrium in groups 1, 2, and 3 (*p* = 0.0001, *p* = 0.0012 and *p* = 0.0001, respectively). Haplotype analyses revealed that there is no linkage disequilibrium between polymorphisms of the analyzed genes.

There was no significant difference in the prevalence of T2DM (*p* = 0.185) or dyslipidemia (*p* = 0.271) between groups, as shown in Table 2. In regards to genetic characteristics, no significant differences between the three groups in terms of polymorphisms for eNOS/*NOS3*, SERCA/*ATP2A2*, Nav1.5/*SCN5A*, Kir6.1/*KCNJ8*, or Kv1.5/*KCNA5* were noticed. However, significant differences (*p* < 0.05) for the SNPs rs5215_GG, and rs5219_AA of Kir6.2/*KCNJ11* were observed, as shown in Table 2. Table 3 displays significant differences between normal subjects (group 3) and patients with either CAD (group 1) or microvascular dysfunction (group 2).

**Table 2:** Clinical and genetic characteristics (ANOVA analysis with Bonferroni correction)

	Coronary artery disease (*n* = 155)	Microvascular dysfunction (*n* = 46)	Normal subjects (*n* = 41)	*p*
Age	66.62 ± 11.935	59.38 ± 14.92	59.25 ± 10.18	0.0001
Male gender	80 % (124/155)	43.4 % (20/46)	34.1 % (14/41)	0.0001
Family history of MI	48.3 % (75/155)	39.1 % (18/46)	19.5 % (8/41)	0.028
Hypertension	78 % (121/155)	54.3 % (25/46)	17 % (7/41)	0.0001
Type II diabetes mellitus	30.3 % (47/155)	13 % (6/46)	34.1 % (14/41)	0.185
Dyslipidemia	51.6 % (80/155)	32.6 % (15/46)	41.4 % (17/41)	0.271
Smoking status	47 % (73/155)	23.9 % (11/46)	24.3 % (10/41)	0.030
eNOS/*NOS3*	–	–	–	NS
SERCA/*ATP2A2*	–	–	–	NS
Nav1.5/*SCN5A*	–	–	–	NS
Kir6.1/*KCNJ8*	–	–	–	NS
Kir6.2/*KCNJ11*: rs5215_GG	12.2 % (19/155)	8.6 % (4/46)	21.9 % (9/41)	0.041
Kir6.2/*KCNJ11*: rs5219_AA	10.9 % (17/155)	10.8 % (5/46)	21.9 % (9/41)	0.019
Kv1.5/*KCNA5*	–	–	–	NS

*NS* not significant results, *p* > 0.050

**Table 3:** Genetic differences comparing with “normal” population

	Normal subjects (group 3) *n* = 41	Coronary artery disease (group 1) *n* = 155	*p*
(a)			
Nav1.5/*SCN5A*: rs1805124_GG	19.5 % (8/41)	7.7 % (12/155)	0.027
Kir6.2/*KCNJ11*: rs5215_GG	21.9 % (9/41)	10.9 % (17/155)	0.048

	Normal subjects (group 3) *n* = 41	Microvascular dysfunction (group 2) *n* = 46	*p*
(b)			
eNOS/*NOS3*: rs1799983_GT	7.3 % (3/41)	26 % (12/46)	0.021
Kir6.2/*KCNJ11*: rs5218_CT	26.8 % (11/41)	45.6 % (21/46)	0.048
Kir6.2/*KCNJ11*: rs5219_AA	21.9 % (9/41)	8.7 % (4/46)	0.049

Analysis of the prevalence of SNPs among normal subjects (group 3) compared to patients with (a). CAD (group 1) and (b) microvascular dysfunction (group 2)

When correcting for other covariates as risk factors, the rs5215_GG genotype of Kir6.2/*KCNJ11* was found to be significantly associated with CAD after multivariate analysis (OR = 0.319, *p* = 0.047, 95 % CI = 0.100–0.991), evidencing a “protective” role of this genotype, as shown in Table 4a. Similarly, a trend that supports this role of Kir6.2/*KCNJ11* was also observed in microvascular dysfunction for rs5219_AA. In contrast, rs1799983_GT for eNOS/*NOS3* was identified as an independent risk factor following multivariate analysis (Table 4b), which agrees with literature findings as described below.

**Table 4:** Multivariate analysis with: (a) CAD as dependent variable and (b) microvascular dysfunction as dependent variable

	*p*	OR	95 % CI	
(a)				
Age	0.002	1.065	1.024	1.109
Male gender	0.001	6.261	2.467	15.891
Family history of MI	0.009	3.625	1.389	9.460
Hypertension	0.011	3.363	1.317	8.587
Type II diabetes mellitus	0.865	2.599	0.255	9.585
Dyslipidemia	0.768	1.956	0.520	1.986
Smoking status	0.012	3.717	1.335	10.346
Kir6.2/*KCNJ11*: rs5215_GG	0.047	0.319	0.100	0.991
(b)				
Age	0.644	0.992	0.959	1.027
Male gender	0.988	1.007	0.428	2.366
Family history of MI	0.209	0.550	0.217	1.396
Hypertension	0.087	1.506	0.215	1.193
Type II diabetes mellitus	0.597	1.351	0.443	4.120
Dyslipidemia	0.065	1.227	0.525	2.896
Smoking status	0.740	1.172	0.457	3.005
eNOS/*NOS3*: rs1799983_GT	0.023	5.234	1.260	8.521
Kir6.2/*KCNJ11*: rs5219_AA	*0.055*	0.260	0.066	1.028

Note that there is a trend toward reduced prevalence of microvascular dysfunction with the presence of SNP rs5219_AA. These data are shown to emphasize the potentially protective association of SNPs in the Kir6.2 subunit against IHD (see text)

## Discussion

### Implications of the present work

This study describes the possible correlation of polymorphisms in genes encoding for CBFR effectors (i.e., ion channels, nitric oxide synthase, and SERCA) with the susceptibility for microcirculation dysfunction and IHD. Our main findings are as follows:

1. A marked HW disequilibrium in the genotypic distribution of rs1799983 polymorphism for eNOS/*NOS3* was observed in all three populations. Moreover, this SNP seems to be an independent risk factor for microvascular dysfunction, as evidenced by multivariate analysis;
2. The SNPs rs5215_GG, rs5218_CT, and rs5219_AA for Kir6.2/*KCJ11* could reduce susceptibility to IHD, since they were present more frequently in patients with anatomically and functionally normal coronary arteries;
3. In particular, with regard to rs5215 for Kir6.2/*KCJ11*, we observed a moderate deviation from the HW equilibrium in the genotypic distribution in the control group. In addition, this genotype appears to be an independent protective factor in the development of IHD, as evidenced by multivariate analysis;
4. Furthermore, the trend observed for the SNP rs5219_AA of Kir6.2/*KCNJ11* may suggest a role for this genotype in protecting against coronary microvascular dysfunction;
5. The rs1805124_GG genotype of Nav1.5/*SCN5A* seems to play a role against CAD;
6. No association seems to exist between the polymorphisms of SERCA/*ATP2A2*, Kir6.1/*KCNJ8*, and Kv1.5/*KCNA5* and the presence IHD;
7. All groups are comparable regarding the cardiovascular risk factors of T2DM and dyslipidemia, illustrating a potentially important implication of genetic polymorphisms in the susceptibility to IHD.

It is important to underline that the control group (group 3) is a high-risk population, because of their cardiovascular risk factors (hypertension = 17 %, T2DM = 34.1 %, dyslipidemia = 41.4 %), with an appropriate indication for coronary angiography, in accordance with current guidelines. Nevertheless, these patients were demonstrated to have both anatomically and functionally normal coronary arteries. Moreover, as shown in Tables 2 and 3, we observed that rs5215_GG, rs5218_CT and rs5219_AA for Kir6.2/*KCNJ11* had a higher prevalence in this group, compared to patients with CAD and patients with microvascular dysfunction. Moreover, as shown in Table 4, the presence of the rs5215_GG polymorphism for the Kir6.2 subunit was inversely correlated with the prevalence of cardiovascular risk factors and CAD, whereas rs5219_AA of the Kir6.2 subunit trended towards an inverse correlation with coronary microvascular dysfunction. On the other hand, the SNP rs1799983_GT of eNOS was confirmed to be an independent risk factor for microvascular dysfunction. Our data suggest that the presence of certain genetic polymorphisms may represent a non-modifiable protective factor that could be used to identify individuals at relatively low-risk for cardiovascular disease, regardless of the presence of T2DM and dyslipidemia.

### Current clinical and research context

In normal coronary arteries, particularly the coronary microcirculation, there are several different mechanisms of CBFR, including endothelial, neural, myogenic, and metabolic mediators [2, 8, 10, 12, 14, 15, 37, 55, 63, 64, 69]. In particular, endothelium-dependent vasodilation acts mainly via eNOS-derived nitric oxide (NO) in response to acetylcholine and shear stress. NO increases intracellular cyclic guanosine monophosphate. It also causes vasodilation via activation of both KCa channels and KATP channels. Recent data suggested a pathophysiologically relevant role for the polymorphisms of eNOS/NOS3 in human coronary vasomotion [40–43]. Our data suggest that rs1799983_GT at exon 7 (Glu298Asp, GAG-GAT) of eNOS/*NOS3* represents an independent risk factor for coronary microvascular dysfunction, which agrees with a recent meta-analysis reporting an association of this SNP with CAD in Asian populations [74]. In addition, this SNP has been associated with endothelial dysfunction, although the mechanisms are not well defined [30]. Consistently, a recent study performed on 60 Indian patients with documented history of CAD reported a significantly higher frequency of rs1799983 (*p* < 0.05) compared to control subjects, indicating that variations in *NOS3* gene may be useful clinical markers of endothelial dysfunction in CAD [54]. Interestingly, another association between rs1799983_GT and impaired collateral development has been observed in patients with a high-grade coronary stenosis or occlusion [19].

As is well known, the significance of the mechanisms of CBFR is partly determined by the location within the coronary vasculature. For instance, for vessels with a diameter of <200 μm—which comprise the coronary microcirculation—metabolic regulation of coronary blood flow is considered the most important mechanism [24, 63]. Importantly, many of these mediators of metabolic regulation act through specific ion channels. In particular, in both coronary artery smooth muscle cells and endothelial cells, potassium channels determine the resting membrane potential (Em) and serve as targets of endogenous and therapeutic vasodilators [9, 27]. Several types of K^+^ channels are expressed in the coronary tree. The KATP channels couple cell metabolic demand to conductance, via pore-forming (Kir6.1 and/or Kir6.2) subunits and regulatory [sulphonylurea-binding (SUR 1, 2A, or 2B)] subunits. Kir6.x allows for channel inhibition by ATP, while SURx is responsible for channel activation by ADP and Mg^2+^. KATP channel activation results in an outward flux of potassium and consequent hyperpolarization, resulting in voltage-gated calcium channel closure, decreased Ca^2+^ influx, and ultimately vasodilation [1, 5, 18, 20, 21, 33, 61, 62, 73, 75].

Our data do not support any significant difference regarding the Kir6.1 subunit of the KATP channel. On the other hand, this study suggests an important role of specific SNPs for the Kir6.2 subunit (Tables 2, 3)—i.e., rs5215, rs5219, and rs5218—in the susceptibility to IHD and microvascular dysfunction. These SNPs are among the most studied KATP channel polymorphisms, especially in the context of diabetes mellitus. In fact, in both Caucasian and Asian populations, these three SNPs as well as other genetic polymorphisms for the *KCNJ11* gene have been associated with diabetes mellitus [34, 35, 44, 50, 57, 58, 70]. Nevertheless, the precise structure–function impacts of the various amino acid substitutions remain unclear. The rs5215 and rs5219 polymorphisms, also known as I337V and E23K, respectively, are highly linked with reported concordance rates between 72 and 100 % [22, 23, 56]. The high concordance between rs5219 and rs5215 suggests that these polymorphisms may have originated in a common ancestor, further indicating a possible evolutionary advantage to their maintenance in the general population [49]. In our study, multivariate analysis suggests both an independent protective role of the rs5215_GG against developing CAD and a trend for rs5219_AA to be associated with protection against coronary microvascular dysfunction (Table 4a, b). The variant rs5215_GG is a missense SNP located in the gene *KCNJ11* at exon 1009 (ATC-GTC) and results in the substitution of isoleucine (I) residue with valine (V) [23]. Future studies are necessary to better understand the influence of this single amino acid variant on the function of the channel.

In humans, vasodilation of the coronary microvasculature in response to hypoxia and KATP channel opening are both impaired in diabetes mellitus [39]. It is also described that gain-of-function mutations of the *KCNJ11* gene cause neonatal diabetes mellitus, and loss-of-function mutations lead to congenital hyperinsulinism [43]. Our study is not discordant with previous studies about the correlation of SNPs of the Kir6.2 subunit and diabetes mellitus. Rather, our findings show that these SNPs are correlated with anatomically and functionally normal coronary arteries, independent of the presence of either diabetes mellitus or dyslipidemia.

These data suggest the possibility that these particular SNPs may identify individuals with decreased risk for coronary microcirculatory dysfunction and IHD, regardless of the presence of T2DM and/or dyslipidemia. However, further studies are necessary to confirm these findings. In this context, to better investigate the implications of genetic variation in the KATP channel, future studies should include ion channel’s functional modification due to the SNPs and analysis of SUR subunits.

More than 40-kV channel subunits have been identified in the heart, and sections of human coronary smooth muscle cells demonstrate Kv1.5 immunoreactivity [16, 17, 27, 38]. Through constant regulation of smooth muscle tone, Kv channels contribute to the control of coronary microvascular resistance [4, 7]. Pharmacologic molecules that inhibit Kv1.5 channels such as pergolide [25], 4-amino-pyridine [32], and correolide [17], lead to coronary smooth muscle cell contraction and block the coupling between cardiac metabolic demand and coronary blood flow. However, no significant differences were identified between the study groups in terms of the particular polymorphisms for Kv1.5 that were analyzed in this study.

Expression of the voltage-dependent Na^+^ channel (Nav) has been demonstrated in coronary microvascular endothelial cells [3, 66]. Our analysis reveals a possible implication of the polymorphism rs1805124_GG for Nav1.5 channel with the presence of anatomically and functionally normal coronary arteries. This SNP leads to a homozygous 1673A-G transition, resulting in a His558-to-Arg (H558R) substitution. It is important to underline that our data are the first to correlate the polymorphism rs1805124_GG with IHD. Further research is necessary to confirm the observed implication.

Finally, we have analyzed the sarco/endoplasmic reticulum calcium transporting Ca^2+^-ATPase (SERCA), which is fundamental in the regulation of intracellular Ca^2+^ concentration [6]. SERCA is an intracellular pump that catalyzes the hydrolysis of ATP coupled with the translocation of calcium from the cytosol into the lumen of the sarcoplasmic reticulum. Although this pump plays a critical role in regulation of the contraction/relaxation cycle, our analysis did not reveal any apparent association between genetic variants of SERCA and the prevalence of microvascular dysfunction or IHD.

## Conclusions

This pilot study is the first to compare the prevalence of SNPs in genes encoding coronary ion channels between patients with CAD or microvascular dysfunction and those with both anatomically and functionally normal coronary arteries. Taken together, these results suggest the possibility of associations between SNPs and IHD and microvascular dysfunction, although the precise manners by which specific genetic polymorphisms affect ion channel function and expression have to be clarified by further research involving larger cohorts.

### Limitations and future perspectives

Notable limitations of this pilot study are as follows:

1. Due to the lack of pre-existing data, the power calculation was performed in advance on the basis of assumptions of allele frequencies and the population at risk.
2. The sample size for each group is small, mainly due to both the difficulty in enrolling patients with normal coronary arteries and normal microvascular function (group 3) and the elevated costs of the supplies such as Doppler flow wires.
3. There is a lack of ethnic diversity of our cohort.
4. Currently, there is an absence of supportive findings in another independent cohort or population. However, our pilot study included patients within a well-defined, specific population and was aimed to identify the presence of statistical associations between selected genetic polymorphisms and the prevalence of a specific disease.
5. There is a lack of functional characterization of the described genetic polymorphisms.
6. We have not identified any correlation between novel SNPs and IHD. Nevertheless, we completely analyzed exon 3 of both KCNJ8 and KCNJ11 genes (Kir6.1 and Kir6.2 subunit, respectively) as well as the whole coding region of KCN5A gene (Kv1.5 channel). Moreover, we examined previously described SNPs since there are no data in the literature regarding the possible association of the prevalences of those polymorphisms in the examined population.

More extensive studies are necessary to confirm our findings, possibly with a larger number of patients. Future investigations are also required to confirm the roles of ion channels in the pathogenesis of coronary microvascular dysfunction and IHD. These studies should involve analysis of both other subunits of the KATP channels (i.e., sulfonylurea receptor, SURx) and further coronary ion channels (e.g., calcium-dependent K channels), as well as in vitro evaluation of ion channel activity by patch clamp and analysis of channel expression in the human cardiac tissue. Moreover, to better address the significance of microvascular dysfunction in IHD, it could be interesting to analyze typical atherosclerosis susceptibility genes (e.g., PPAP2B, ICAM1, et al.).
